# Is ABCC6 a genuine mitochondrial protein?

**DOI:** 10.1186/1756-0500-6-427

**Published:** 2013-10-23

**Authors:** Marc Ferré, Pascal Reynier, Arnaud Chevrollier, Delphine Prunier-Mirebeau, Georges Lefthériotis, Daniel Henrion, Dominique Bonneau, Vincent Procaccio, Ludovic Martin

**Affiliations:** 1 CNRS 6214/INSERM 1083, Angers University, Angers, France; 2 Department of Biochemistry and Genetics, University Hospital, Angers, France; 3 PXE Consultation Center, University Hospital, Angers, France

**Keywords:** Mitochondria, ABCC6/MRP6, Pseudoxanthoma elasticum (PXE), Mitochondria-associated membrane (MAM)

## Abstract

**Background:**

A recent article in *Circulation Research* suggests that the protein ABCC6, which when defective is responsible for pseudoxanthoma elasticum, an inherited condition with skin, eye and cardiovascular manifestations, is associated with dysfunction in mitochondria – Martin et al.: **ABCC6 Localizes to the Mitochondria-Associated Membrane*.****Circ Res* 2012, **111:**516**–**520. We present complementary information based on a bioinformatics analysis, which was not performed in the article cited, to examine the suggestion that ABCC6 is localized to mitochondria.

**Results:**

All the computational strategies and integrative approaches that constitute references in the field indicate that ABCC6 is localized outside of mitochondria.

**Conclusion:**

Our computational and integrative results, including both experimental and predictive data, show that there is no support in favor of the localization of ABCC6 in mitochondria.

## Discussion

Pseudoxanthoma elasticum (PXE), an inherited condition with skin, eye and cardiovascular manifestations resulting from dystrophic calcification, is caused by a defect in the ATP-binding cassette, subfamily C, member 6 protein (ABCC6) [1]. ABCC6 and the murine ortholog Abcc6, which have been repeatedly located to the plasma membrane of the liver and proximal tubule kidney cells, are virtually absent in affected tissues in PXE and in related murine conditions [1-3]. These findings are of major pathophysiological significance since they indicate that ABCC6 mediates remote calcification *via* the circulation and that PXE is a systemic condition [4]. However, a recent article in *Circulation Research* claiming that ABCC6 localizes to the mitochondria-associated membrane (MAM) [5] has given rise to some debate [6,7]. MAMs have been defined as the contact sites between mitochondria and the ER with no involvement of any direct membrane fusion [8]. In their article, the authors describe various structural mitochondrial changes in *Abcc6−/−* kidney and heart cells, though not in liver cells, as well as changes in the endoplasmic reticulum (ER) bordering mitochondria and decreased reserve respiration capacity in liver cells. Taken together, these findings may suggest dysfunction in mitochondria rather than in MAM. We therefore set out to test the possibility of ABCC6 being localized to mitochondria, using the computational and integrative approaches that are now references in the field.

### Computational analysis

Mitochondrial proteins may be targeted to the mitochondrial matrix, inner membrane, outer membrane, or the intermembrane space [9]. Proteins destined for import into the organelle *via* a cleavable peptide presequence, typically 15–50 residues long, contain an α-helix with positively charged residues on one side, and uncharged hydrophobic residues on the other. Some of these proteins are subsequently directed to the inner membrane and the intermembrane space according to the consecutive peptide sequences they carry.

The identification of the targeting sequence of mitochondrial proteins by means of a purely computational strategy is now well established and commonly used. Several algorithms have been developed for the detection of mitochondrial targeting sequences on proteins translated in the cytosol. Although these tools suffer from the limitation of leading to false-positive predictions of mitochondrial localization, they all predict the presence of ABCC6 outside mitochondria and the ER. Thus:

Predotar [10] localizes ABCC6 neither in mitochondria nor the endoplasmic reticulum (*p*=0.99);

MitoProt [11] finds a low probability of the protein being exported to mitochondria (*p*=0.0304);

TargetP [12] indicates a location other than mitochondria and the secretory pathway (score: 0.845);

PProwler [13] localizes the protein neither in mitochondria, the secretory pathway, nor the peroxisomal matrix (score: 0.59);

CELLO [14], WoLF PSORT [15] and PSORT-II [16] localize the protein in the plasma membrane; and

iPSORT [17] predicts the protein has no signaling or mitochondrial targeting peptide.

In addition, some localization software techniques [14-16], which rely not only on the N-terminal targeting sequence but apply to whole-cell localizations, predicts the localization of ABCC6 in the plasma membrane.

### Integrative analysis

Currently, the most successful experimental strategy for establishing mitochondrial protein localization combines in-depth protein mass spectrometry of 14 mouse tissue samples, large-scale green fluorescent protein-tagging microscopy, machine learning, literature curation, the presence of mitochondrial-specific protein domains, mRNA coexpression, targeting-signal prediction, transcriptional induction during mitochondrial proliferation, homology to yeast mitochondrial proteins, and protein homology to *Rickettsia*, a relative of the mitochondrion’s bacterial ancestor [18]. The results of all these integrative approaches, compiled in the online databases MitoP2 [19], MitoCarta [20], MitoProteome [21], MitoMiner [22], and HMPDb [23], indicate that ABCC6 is not localized in mitochondria.

The most comprehensive mitochondrial database is now estimated to be 85 % complete, containing about 1,100 mitochondrial proteins [20]. The incompleteness may be explained by the fact that the protein composition of mitochondria varies from one tissue to another. Approximately half of the mitochondrial proteins are ubiquitous, whereas the expression of the remainder is tissue-specific expression [20,24]. However, Martin *et al.*[5] found ABCC6 localized to mitochondria in the liver as well as the kidney, both organs that were studied in the integrative works cited above.

## Conclusions

As emphasized by Martin *et al.*[5], the determination of the subcellular location of ABCC6 is critical in deciphering the exact role of the transporter in physiology, in general, and in the pathophysiology of PXE, in particular, when ABCC6 is absent or defective. Biochemical and immunological approaches have led to the description of ABCC6 as a cytoplasmic membrane protein. Our computational and integrative results show that there is no support in favor of the localization of ABCC6 either in mitochondria or in the ER membrane.

We hypothesize that the MAM localization of ABCC6 may be transitory. It would be interesting to study the vesicular traffic through the sites of contact between mitochondria and the ER, especially as it has been recently shown that the plasma membrane contributes in part to the lipid bilayers of the autophagosome, creating a possible pathway requiring the close contact between ABCC6 and mitochondria during mitophagy [25].

## Abbreviations

ER: Endoplasmic reticulum; MAM: Mitochondria-associated membrane; PXE: Pseudoxanthoma elasticum.

## Competing interests

The authors state no conflict of interest.

## Authors’ contribution

MF, PR, & LM initiated the study. MF conducted the experiments. MF & AC drafted the article. MF wrote the final manuscript. AC, PR, DPM, GL, DH, DB, VP, & LM provided critical revision. All authors read and approved the final manuscript.

## Authors’ information

MF obtained his Ph. D. (2009) with the bioinformatics analysis of the mitochondrial proteome.

MF, AC, PR, DPM, DB, & VP belong to Mitolab (http://mitolab.eu), a French academic team of the CNRS 6214/INSERM 1083 research unit specialized in disorders involving mitochondrial dynamics and bioenergetics. The Department of Integrated Neurovascular and Mitochondrial Biology (CNRS 6214/INSERM 1083) is headed by DH.

PR, DB, & VP are members of the French Reference Centre for Mitochondrial Diseases in children and adults.

GL & LM are members of the PXE Consultation Center of the University Hospital of Angers, France.
